# A screen for sleep and starvation resistance identifies a wake-promoting role for the auxiliary channel unc79

**DOI:** 10.1093/g3journal/jkab199

**Published:** 2021-06-16

**Authors:** Kazuma Murakami, Justin Palermo, Bethany A Stanhope, Allen G Gibbs, Alex C Keene

**Affiliations:** 1 Department of Biological Sciences, Florida Atlantic University, Jupiter, FL 33458, USA; 2 Department of Biological Sciences, University of Nevada, Las Vegas, Las Vegas, NV 89154, USA

**Keywords:** sleep, metabolic rate, *Drosophila*, feeding, energy stores, mushroom body

## Abstract

The regulation of sleep and metabolism are highly interconnected, and dysregulation of sleep is linked to metabolic diseases that include obesity, diabetes, and heart disease. Furthermore, both acute and long-term changes in diet potently impact sleep duration and quality. To identify novel factors that modulate interactions between sleep and metabolic state, we performed a genetic screen for their roles in regulating sleep duration, starvation resistance, and starvation-dependent modulation of sleep. This screen identified a number of genes with potential roles in regulating sleep, metabolism, or both processes. One such gene encodes the auxiliary ion channel UNC79, which was implicated in both the regulation of sleep and starvation resistance. Genetic knockdown or mutation of *unc79* results in flies with increased sleep duration, as well as increased starvation resistance. Previous findings have shown that *unc79* is required in pacemaker for 24-hours circadian rhythms. Here, we find that *unc79* functions in the mushroom body, but not pacemaker neurons, to regulate sleep duration and starvation resistance. Together, these findings reveal spatially localized separable functions of *unc79* in the regulation of circadian behavior, sleep, and metabolic function.

## Introduction

Sleep acutely regulates metabolic function, and growing evidence suggests that these processes interact to regulate many biological functions including cognition, physiology, and longevity (Hartmann 1974; Siegel 2005; Joiner 2016; Beckwith and French 2019). At the clinical level, many diseases related to metabolic dysfunction including diabetes, heart disease, and obesity are associated with chronic sleep loss (Taheri *et al.* 2004; Arble *et al.* 2015; Reutrakul and Van Cauter 2018). In addition, diet potently influences sleep duration and quality, indicating that neural systems regulating sleep are sensitive to internal nutrient stores and food availability (Catterson *et al.* 2010; Grandner *et al.* 2010, 2014; Linford *et al.* 2012). Identifying how sleep, diet, and metabolic regulation are interconnected is critical to understanding the fundamental functions of sleep.

Interactions between sleep, feeding, and metabolic regulation are highly conserved between the fruit fly and mammals (Griffith 2013; Yurgel *et al.* 2015; Beckwith and French 2019; Shafer and Keene 2021). Experimental evolution and artificial selection approaches have revealed a relationship between sleep, feeding, and starvation resistance (Masek *et al.* 2014; Slocumb *et al.* 2015; Brown *et al.* 2019). For example, selection for short-sleeping flies results in reduced energy stores and sensitivity to starvation, while selecting for starvation resistance increases sleep duration (Seugnet *et al.* 2009; Masek *et al.* 2014; Slocumb *et al.* 2015). Furthermore, examining naturally occurring genetic variation in sleep and starvation resistance in *Drosophila melanogaster* from different geographic localities suggests sleep and starvation resistance are inversely related (Brown *et al.* 2018; Sarikaya *et al.* 2020). In addition, shared neural circuits appear to regulate both feeding and starvation resistance, including a role for Insulin-like peptides and the Leucokinin Receptor, and naturally occurring variation in levels of the *foraging* gene impact feeding and starvation resistance (Thimgan *et al.* 2012; Allen *et al.* 2017; Zandawala *et al.* 2018). The interactions between these traits under conditions of experimental evolution raise the possibility that shared genetic factors underlie sleep and starvation resistance.

Energy conservation has long been proposed to be primary function of sleep (Hartmann 1973; Berger and Phillips 1995). *Drosophila* live for only a few days in the absence of food, providing an excellent model to examine the effects of sleep on metabolic regulation and energy conservation (Yurgel *et al.* 2015; Ly *et al.* 2018). Quantifying longevity under starvation conditions provides a readout of overall energy stores and metabolic rate (Baldal *et al.* 2006; Schwasinger-Schmidt *et al.* 2012). In addition, flies acutely suppress sleep and increase activity in response to starvation, providing a system to investigate acute modulation of sleep and metabolic function (Lee and Park 2004; Keene *et al.* 2010). Genetic screens and genomic analyses have identified many regulators of sleep, metabolic regulation, and starvation resistance, establishing flies as a model for studying the interactions between these processes (Harbison *et al.* 2005; Jumbo-Lucioni *et al.* 2010; Murakami *et al.* 2016; Sonn *et al.* 2018). Many of the genes initially identified through screening for short-sleeping mutants have reduced life spans or increased sensitivity to stressors, though the relationship with starvation resistance is less clear (Koh *et al.* 2008; Bushey *et al.* 2010; Hill *et al.* 2018). A complete understanding of how these processes are integrated requires the localization of genes and neurons that regulate sleep and metabolic processes.

The study of sleep in flies has predominantly focused on the role of genes and neurons under fed conditions, leading to the identification of many distinct circuits that promote sleep and wakefulness (Allada and Siegel 2008; Sehgal and Mignot 2011; Ly *et al.* 2018). There is growing evidence that additional cell types are critical regulators of sleep including multiple classes of glia, endocrine cells, and the fat body (Artiushin *et al.* 2018; Stahl *et al.* 2018; Vanderheyden *et al.* 2018; Yurgel *et al.* 2018; Ertekin *et al.* 2020). Furthermore, the genes and neurons regulating sleep can differ based on environmental context (Griffith 2013; Beckwith and French 2019; Shafer and Keene 2021). These studies highlight brain-periphery interactions that are change in response numerous environmental contexts including food availability. Therefore, identifying genetic regulators that impact both sleep and metabolic function requires investigating both neuronal and nonneuronal cell types.

Here, we have performed a genetic screen to identify genetic regulators of sleep and metabolic function, targeting genes ubiquitously to identify factors that function both within the brain and the periphery. Flies were tested in a pipeline that measured sleep parameters under fed and starved conditions, followed by assessment of starvation resistance. This screen identified several candidate genes regulating sleep and metabolic function including the sodium leak channel *NALCN* accessory subunit *uncoordinated 79* (*unc79*). The gene was first identified in the nematode *Caenorhabditis elegans* in a screen that identified numerous regulators behavior and morphology (Brenner 1974). Both *unc79* and its partner *unc80*, are auxiliary subunits of r the drosophila *Narrow Abdomen* (*na*), that is a critical regulator of the circadian clock in *Drosophila* (Lear *et al.* 2005, 2013; Flourakis *et al.* 2015). Flies mutant for *na* mutants have altered response to anesthetics in these behavioral assays, deficits in circadian locomotor rhythms, and altered social clustering (Humphrey *et al.* 2007; Burg *et al.* 2013). Here, we find additional roles for *unc79* in regulating sleep and metabolic regulation. Furthermore, we provide evidence that *unc79* regulates these processes through independent neural circuit and molecular mechanism from those that govern circadian rhythms.

## Methods

### Fly husbandry

Flies for behavioral experiments were maintained and tested in humidified incubators at 25°C and under 65% humidity (Powers Scientific). Flies were reared on a 12 hours: 12 hours light–dark cycle for experiments prior to behavioral analysis. All flies were maintained on Nutri-fly Drosophila food (Genesee Scientific). All RNAi lines tested were obtained from the Bloomington *Drosophila* Stock Center (Bloomington, IN, USA) (Ni *et al.* 2008; Perkins *et al.* 2015) and the Vienna *Drosophila* Resource Center stock (# 45780) was used to validate RNAi phenotypes from the initial screen (Vienna, Austria) (Dietzl *et al.* 2007) (see Table 1). Bridget Lear (Northwestern) generously provided *unc79*^F01615^ and *unc79*^F01615^ lines (Lear *et al.* 2013).

**Table 1:** Circadian phenotypes in unc79 knockdown flies. Data depict the power and period (length) of rhythms in unc79 knockdown and associated control lines.

Name	Power	±	Period (hours)	±	*N*
W^1118^ (+)	145.5	18.38	24.78	0.1326	32
*luc* ^RNAi^/+	418	38.99	24.24	0.09269	32
*unc*79^RNAi^/+	207.7	22.14	23.84	0.04861	32
nSyb^G4L4^>*luc*^RNAi^	420.4	56.79	25.58	0.02795	32
nSyb ^G4L4^>*unc*79^RNAi^	45.4	7.049	26.32	0.9583	31
OK107 ^G4L4^>*luc*^RNAi^	309.8	38.95	23.54	0.09379	32
OK107 ^G4L4^*>unc*79^RNAi^	129	13.16	23.58	0.1117	30

### Behavioral analysis

*Drosophila* Activity Monitors (DAM; Trikinetics, Waltham, MA, USA) were used for all behavioral analyses. The DAM system detects activity by monitoring infrared beam crossings for each animal (Pfeiffenberger *et al.* 2010a). These data were used to calculate sleep information by extracting immobility bouts of 5 minutes using the Drosophila Sleep Counting Macro (Pfeiffenberger *et al.* 2010b). All behavioral experiments used 5–7 days old mated female flies unless otherwise noted. We have previously found that females display more robust starvation-induced sleep suppression, and have used mated females in a prior genetic screen (Keene *et al.* 2010; Murakami *et al.* 2016). For experiments examining the effects of starvation on sleep, activity was then measured for 24 hours on food, prior to transferring flies into tubes containing 1% agar (Fisher Scientific) at ZT0 and activity was recorded for an additional 48 hours.

To measure starvation resistance, flies were starved on experimental day 2 by transferring them from food 1% agar (Fisher Scientific) individual DAM tubes containing 1% agar. Activity across the first 48 hours on agar was used to measure starvation-induced sleep suppression as previously described (Masek *et al.* 2014). While previous studies measured starvation-induced sleep suppression on day 1 of starvation, we measured this process for multiple days (Keene *et al.* 2010). To measure starvation resistance, activity was then recorded until death. Death was manually determined as the last activity time point from the final recorded activity bout for each individual fly.

For experiments quantifying circadian rhythm analysis, locomotor activity under free-running conditions was measured using the DAM system as previously described (Chiu *et al.* 2010). Individual flies were housed in 10% sucrose DAM tubes instead of standard fly food to prevent larval development that interferes that the circadian assay. Five-day-old adult flies were entrained to light-dark (LD) 12 hours: 12 hours (12:12) cycles for three days, then transferred to constant darkness (DD) for 7–8 days. Locomotor activity data were analyzed using Clocklab software (ActiMetrics, Version 2.72). Individual periods were calculated from 7 to 8 days activity data during DD using chi-square periodogram. Rhythm strength was determined by Fast Fourier Transform (FFT) analysis as previously described (Chiu *et al.* 2010).

### Statistical analysis

Statistical analyses were performed using InStat software (GraphPad Software 6.0). For analysis of sleep, we employed a one- or two-way ANOVA followed by a Tukey’s *post hoc* test. For starvation resistance, we applied Kaplan–Meier analysis by grouping each genotype.

### Data availability

Fully analyzed data and statistics are available as supplementary files. Supplementary material is available at figshare: https://www.doi.org/10.25387/g3.14738895. Raw data will be made available upon request.

## Results

**Figure 1:**
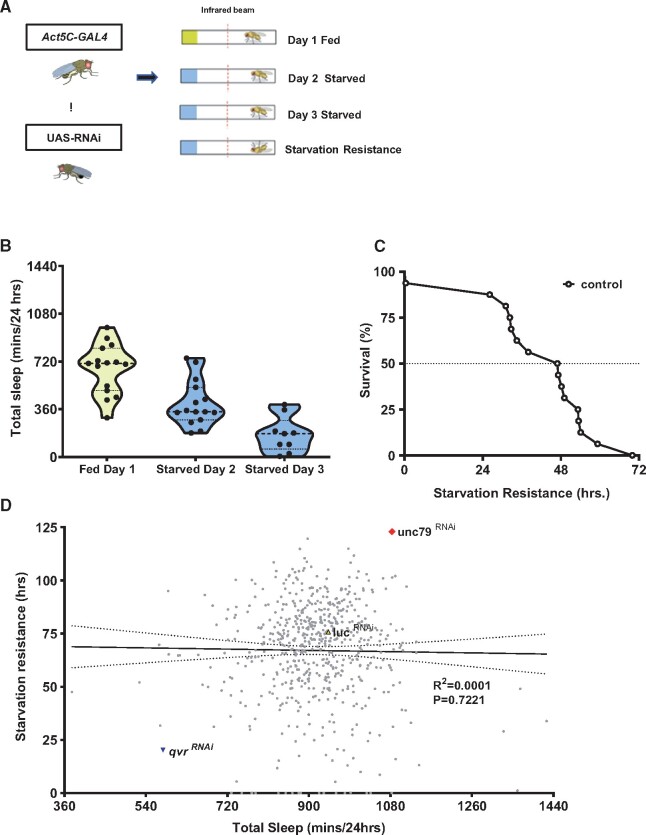
Screening for sleep and starvation resistance. (A) Schematic of TRiP Screen. Ubiquitous Act5c GAL4 driver is crossed to TRiP lines, F1 flies were transferred to *Drosophila* Activity Monitor tubes. Sleep was measured for 24 hours on standard food, after which, flies were transferred to agar where they remained until death to quantify starvation-induced sleep suppression and starvation resistance. (B) Control *w^1118^* flies suppressed sleep during the first day of starvation, and an even greater suppression was observed on day two of starvation. (C) *w^1118^* flies survived approximately 3 days on agar, providing a robust readout sleep and starvation resistance. Total sleep (minutes) is measured over 24 hours period and starvation resistance is measured in hours. (D) Scatter plot for fed total sleep on *x*-axis plotted (minutes) to starvation resistance on *y*-axis (hours) [Simple Linear Regression: *F*_(1, 609)_ = 0.1265, *R*^2^ = 0.0001, *P* > 0.7223, *N* = 616 fly lines). Control flies with no endogenous targets Act5c GAL4 drive luc ^RNAi^ (yellow), lines tested in (gray), qvr has the lowest sleep and SR (blue), and unc79 highest sleep and SR (red).

We developed a pipeline that measured sleep, starvation-induced sleep suppression, and starvation resistance in individual flies. Sleep was measured for 24 hours on standard food, after which, flies were transferred to agar where they were maintained until death to measure starvation-induced sleep suppression and starvation resistance (Figure 1A). We first validated the pipeline in *w^1118^* flies, and found that flies robustly reduce sleep the first 24 hours of starvation and live an average of 48 hours without food (Figure 1C).

To screen for novel regulators of sleep and starvation resistance we ubiquitously knocked down genes by expressing RNAi transgenes from the TRiP Collection under control of the *Actin5C*-GAL4 driver (Perkins *et al.* 2015). Control flies harboring *Actin5C*-GAL4 driving UAS-*luciferase*-RNAi (*Act5c*>*Luc ^RNAi^*), a control with no endogenous targets, flies suppressed sleep during the first day of starvation, and an even greater suppression was observed on day 2 of starvation. To enrich for genes that may be involved in sleep or metabolic function, candidate genes were selected from a genome-wide analysis of polymorphisms and genomic markers of selection in flies selectively bred for starvation resistance (Hardy *et al.* 2018). Of the 1429 significant genes from this analysis, we identified 914 genes that TRiP RNAi stocks available (Perkins *et al.* 2015; Hardy *et al.* 2018). Of these, 299 lines (32.7%) were lethal with ubiquitous knockdown and, therefore, were not screened. In total, we screened 616 lines for sleep, starvation-induced sleep suppression (Figure 1D and Supplementary Table S1). Ubiquitous knockdown of the previously identified Ly-6 transmembrane protein, *qvr/sleepless*, resulted in the shortest sleep duration, confirming the ability of the screening procedure to effectively identify genetic regulators of sleep (Koh *et al.* 2008). To examine the relationship between sleep and starvation resistance we plotted the average for each trait. There was no association between these traits (*r*^2^ < 0.001, *P* > 0.772), suggesting sleep and starvation resistance independently regulated (Figure 1D). However, we identified a number of genes where ubiquitous knockdown resulted in increased sleep on food and greater starvation resistance (Figure 1D). We also examined the correlation between genes screened for different sleep parameters. For example, daytime sleep duration is correlated with nighttime sleep duration, suggesting shared genes regulate both processes (Supplementary Figure S1A). We found average bout length was inversely correlated with sleep bout number, suggesting these traits are functionally related (Supplementary Figure S1B). However, no correlation was observed between waking activity and total sleep (Supplementary Figure S1C) suggesting independent regulation of these traits. We chose to focus on the gene encoding for the NALCN auxiliary protein, *unc79* because of the robustness of each phenotype and its role as an essential regulator of circadian rhythms sleep regulation (Lear *et al.* 2005; Joiner *et al.* 2013).

**Figure 2:**
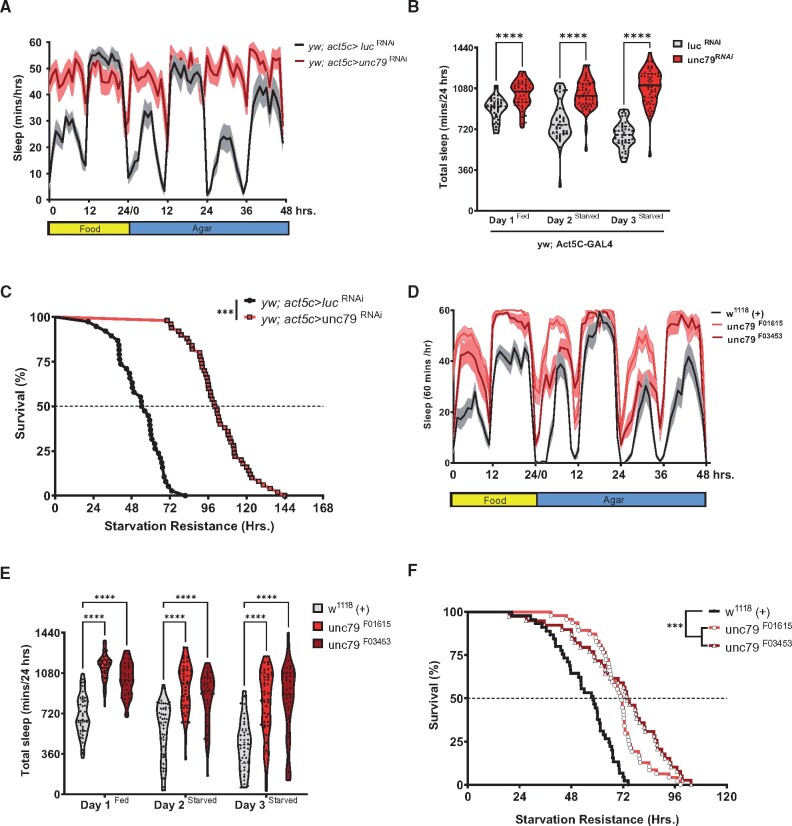
unc79 RNAi and mutants have increased sleep and starvation resistance. (A) Sleep profiles depicting the average sleep each hour over a 72 hours experiment for Act5c>luc ^RNAi^ (gray) and Act5c>*unc79*^RNAi^ (red). Flies were on food for day 1, then transferred to agar for days 2 and 3. (B) Act5c>unc79 ^RNAi^ (red) during fed [two-way ANOVA: *F*_(2, 261)_ = 8.551, *P* < 0.0001 *N* > 39], starved day 1 [two-way ANOVA: *F*_(2, 261)_ = 8.551, *P* < 0.0001, *N* > 39] and starved day 2 [two-way ANOVA: *F*_(2, 261)_ = 8.551, *P* < 0.0001 *N* > 39] flies slept significantly longer compared to Act5c>luc ^RNAi^ (gray) controls. (C) Starvation resistance of Act5c>unc79^RNAi^ (red) is significantly higher than Act5c>luc^RNAi^ (black) control (Gehan-Breslow-Wilcoxon test: χ^2^ = 94.42, *df* = 1, *P* < 0.0001). (D) Sleep profile for hourly sleep averages over a 72 hours experiment for w^1118^, *unc79*^F01615^ (red) and *unc79*^F03453^ (maroon) flies are on food for day 1, then transferred to agar for days 2 and 3. (E) Total sleep is greater in *unc79*^F03453^ mutant under fed [maroon, *F*_(2, 651)_ = 71.46, *P* < 0.0001, *N* ≥ 39], starved day 1 (*P* < 0.001; *N* ≥ 39) and starved day 2 (*P* < 0.001; *N* ≥ 39) conditions compared to control (gray). Total sleep is greater in *unc79*^F01615^ mutant (red) under fed [*F*_(2, 651)_ = 71.46, *P* < 0.0001, *N* ≥ 39], starved day 1 (*P* < 0.001; *N* ≥ 39), and starved day 2 (*P* < 0.001; *N* ≥ 39) conditions compared to w^1118^ control (gray). (F) Starvation resistance is greater in *unc79*^F03453^ (maroon, Gehan-Breslow-Wilcoxon test: χ^2^ = 29.1, *df* = 1, *P* < 0.0001) and *unc79*^F01615^ (red, Gehan-Breslow-Wilcoxon test: χ^2^ = 18.6, *df* = 1, *P* < 0.0001) flied compared to w^1118^ control (gray). All sleep data are violin plots and SR data are survival curves. ***P < 0.001; ****P < 0.0001.

To validate the sleep and starvation phenotypes associated with *unc79* we repeated experiments and examined the sleep profile. Flies with ubiquitous knockdown of *unc79* (*Act5c*>*unc79*^RNAi^) slept significantly more than control flies (Figure 2, A and B). Furthermore, while both control groups suppressed sleep during day 1 and 2 of starvation, *Act5c*>*unc*79^RNAi^ flies did not suppress sleep, suggesting that *unc79* is required for metabolic regulation of sleep (Figure 2, A and B). In addition, starvation resistance was significantly increased in *Act5c*>unc79^RNAi^ flies compared to *Act5c*>*Luc*^RNAi^ controls (Figure 2C). In agreement with our previous findings, waking activity in female control flies increased starvation, but was unchanged in *Act5C*>*Unc79*^RNAi^ flies (Supplementary Figure S2A) (Keene *et al.* 2010). Furthermore, the overall waking activity was elevated in *Act5c*>*Unc79*^RNAi^ flies compared to *Act5c*>*Luc*^RNAi^ controls under fed conditions suggestions that the increased sleep in flies deficient for *unc79* is not due to general lethargy.

To confirm that the observed phenotypes are not due to RNAi off targets we tested flies with a genetic mutation in the *unc79* locus. The independent Pbac element insertions in the *unc79* locus slept longer on food and failed to suppress sleep when starved, phenocopying RNAi knockdown (Figure 2, D and E, Lear *et al.* 2013). Furthermore, *unc79* mutants (*unc79^F03453^* and *unc79^F01615^*) survived significantly longer on agar than respective controls (Figure 2F). Analysis of flies heterozygous for the mutation revealed the long sleeping phenotype is semi-dominant (Figure 2, D–F).

We also assessed male flies to determine whether these phenotypes generalize across sexes. Male *unc79* mutants (*unc79^F03453^* and *unc79^F01615^*) flies slept longer on food and failed to suppress sleep when starved (Supplementary Figure S2A) and survived longer on agar than respective controls (Supplementary Figure S2B), but the response was attenuated compared to female flies. Waking activity in male flies did not differ between fed and starved groups of *unc79* mutants, while controls increase waking activity (Supplementary Figure S2C). Therefore, ubiquitous RNAi knockdown or genetic mutation of *unc79* results in increased sleep and starvation resistance, and impaired metabolic regulation of sleep.

**Figure 3:**
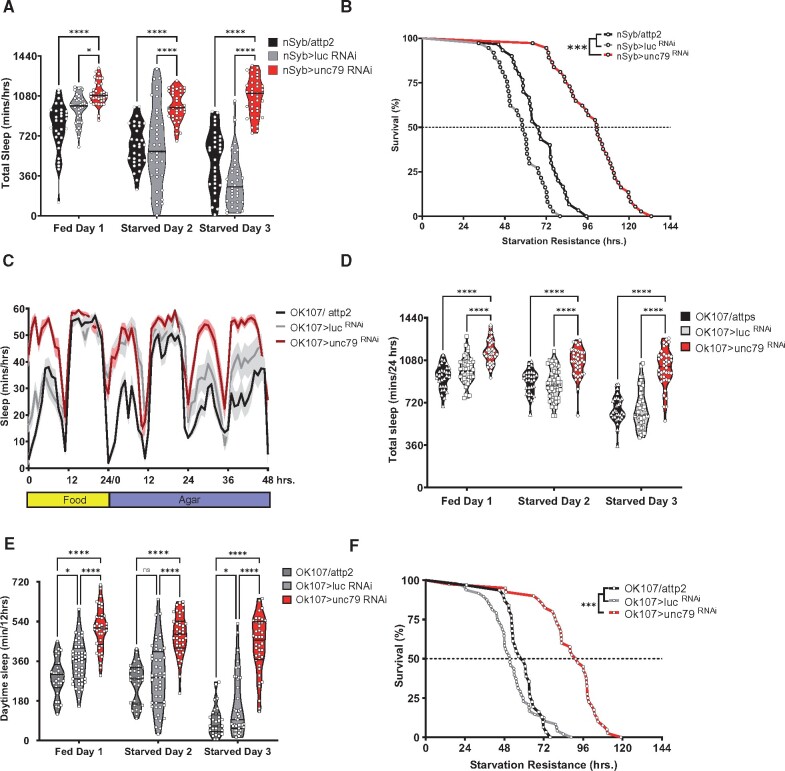
Localization of sleep and starvation resistance phenotype to mushroom body. (A) Pan-neuronal knockdown of unc79 (nSyb>unc79 ^RNAi^, red) is significantly increased in sleep during fed [two-Way ANOVA: *F*_(2, 300)_ = 57.82, *P* < 0.0001, *N* > 30], starved day 1 (*P* < 0.0001, *N* > 30), and starved day 2 (*P* < 0.0001, *N* > 30); while nSyb>attp2 (light gray) and nSyb>luc RNAi controls shows starvation-induced sleep suppression. (B) Starvation resistance for pan-neuronal knockdown of unc79 nSyb>*unc79*^RNAi^ (red) is significantly increased compared to nSyb>attp2 (gray, Gehan-Breslow-Wilcoxon test: χ^2^ = 42, *df* = 1, *P* < 0.0001, *N* > 30) and nSyb>luc RNAi (light gray, Gehan-Breslow-Wilcoxon test: χ^2^ = 64.6, *df* = 1, *P* < 0.0001, *N* > 37) control flies. (C) Sleep profile hourly sleep averages over a 72-hours experiment for mushroom body knockdown of unc79. Flies are on food for day 1, then transferred to agar for days 2 and 3. (D) Mushroom body knockdown of unc79 (OK107>unc79 ^RNAi^, red) is significantly increased in sleep during fed [two-way ANOVA: *F*_(2, 300)_ = 57.82, *P* < 0.0001, *N* > 30] starved day 1 (*P* < 0.0001, *N* > 31), and starved day 2 (*P* < 0.0001, *N* > 31); while Ok107>attp2 (light gray) and Ok107>luc RNAi controls shows starvation-induced sleep suppression. (E) Day-time sleep in flies with mushroom body knockdown of unc79 (OK107>unc79 ^RNAi^, red) is significantly increased under fed conditions [two-way ANOVA: *F*_(2, 348)_ = 43.42, *P* < 0.0001, *N* > 30), starved day 1 (*P* < 0.0001, *N* > 31), and starved day 2 (*P* < 0.0001, *N* > 31); while OK107>attp2 (gray) and OK107>luc ^RNAi^ (light gray) controls maintain normal daytime sleep. (F) Starvation resistance is increased in OK107>*unc79*^RNAi^ flies compared to Ok107>attp2 (gray, Gehan-Breslow-Wilcoxon test: χ^2^ = 47.6, *df* = 1, *P* < 0.0001, *N* > 31) and OK107>*luc*^RNAi^ (light gray, Gehan-Breslow-Wilcoxon test: χ^2^ = 50.5, *df* = 1, *P* < 0.0001, *N* > 31) control flies. All sleep data represent violin plots and SR data are survival curves. *P < 0.05; ***P < 0.001; ****P < 0.0001.

To localize *unc79* function in metabolism and sleep we first targeted *unc79*^RNAi^ to all neurons using the driver n-synaptobrevin-GAL4 (*nsyb*-GAL4) (Riabinina *et al.* 2015). Knockdown in neurons led to flies that slept significantly more than background controls harboring expressing RNAi to luciferase under fed conditions (Figure 3A). Furthermore, flies with pan-neuronal knockdown of *unc79* (*nSyb*-GAL4>*unc79*^RNAi^) also did not significantly reduce sleep during starvation and survived significant longer, suggesting *unc79* functions in neurons to regulate sleep and starvation resistance (Figure 3, A and B). To further localize the function of *unc79* we targeted RNAi to six types of neurons known to modulate sleep. Knockdown in the circadian neurons using *Pdf*-GAL4 or *Tim*-GAL4 did not affect sleep or starvation resistance, suggesting the effects on sleep and metabolic function are independent of its role in circadian activity (Supplementary Figure S3A). Furthermore, no effect was observed knocking down *unc79* in the sleep promoting central complex (*23E10*-GAL4) or broad classes of peptidergic cells (*C929*-GAL4) (Supplementary Figure S3, A–D). Knockdown of *unc79* selectively in the mushroom bodies (*OK107*-GAL4) increased total sleep, specifically during the day, and resulted in increased starvation resistance (Figure 3, C–F). Therefore, selective knockdown with *OK107*-GAL4 largely phenocopies ubiquitous knockdown, raising the possibility that *unc79* functions in the mushroom body to regulate sleep and starvation resistance.

**Figure 4:**
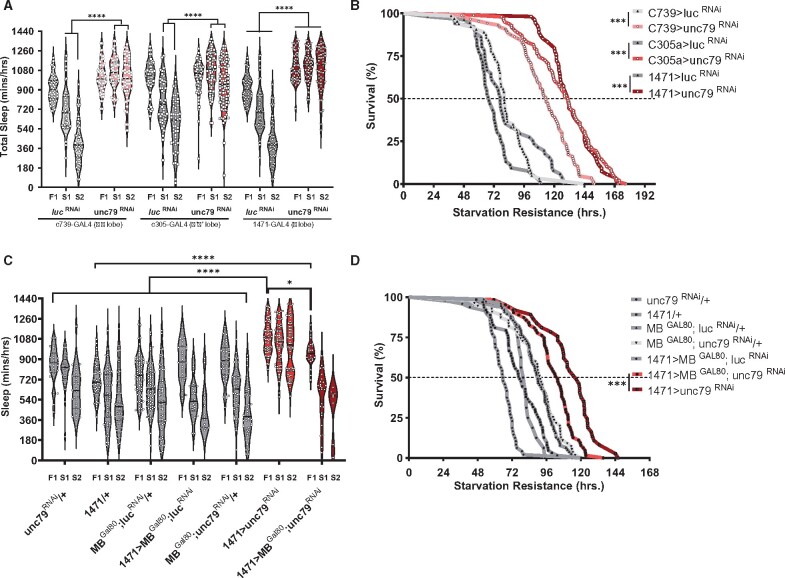
*unc79* function in the mushroom body γ-lobe to regulate sleep and starvation resistance. (A) Flies with *unc79* knocked down in the αβ lobes (c739>unc79 ^RNAi^, pink) fail to suppress starvation during starved day 1 [two-way ANOVA *F*_(10, 1075)_ = 28.56, *P* < 0.0001, *N* > 53] and starved day 2 (*P* < 0.0001) compared to control (c739>luc ^RNAi^, light gray); while fed day 1 did not differ (*P* > 0.1587). Mushroom body α’β’ knockdown of unc79 (c305a>unc79 ^RNAi^, red) fails to suppress starvation during starved day 1 [two-way ANOVA *F*_(10, 1075)_ = 28.56, *P* < 0.0001, *N* > 51] and starved day 2 (*P* < 0.0001) compared to control (c305a>luc ^RNAi^, light gray); while fed day 1 did not differ (*P* > 0.999). Mushroom body γ knockdown of unc79 (1471>unc79 ^RNAi^, maroon) significantly increase total sleep during fed day [two-way ANOVA *F*_(10, 1075)_ = 28.56, *P* < 0.0001, *N* > 53] and fails to suppress sleep on starved day 1 (*P* < 0.0001), and starved day 2 (*P* < 0.0001) compared to control (1471>luc ^RNAi^, dark gray). (B) Starvation resistance increased when mushroom body αβ knockdown of unc79 (c739>unc79 ^RNAi^, pink) is significant (Gehan-Breslow-Wilcoxon test: χ^2^ = 71.36, *df* = 1, *P* < 0.0001, *N* > 63) compared to control (c739>luc ^RNAi^, light gray). Starvation resistance increased when mushroom body α’β’ knockdown of unc79 (c305a>unc79 ^RNAi^, red) is significant (Gehan-Breslow-Wilcoxon test: χ^2^ = 90.45, *df* = 1, *P* < 0.0001, *N* > 51) compared to control (c305a>luc ^RNAi^, gray). Mushroom body γ knockdown of unc79 (1471>unc79 ^RNAi^, maroon) is significant (Gehan-Breslow-Wilcoxon test: χ^2^ = 124.6, *df* = 1, *P* < 0.0001, *N* > 53) compared to control (1471>luc ^RNAi^, dark gray). (C) Mushroom body GAL80 rescue γ knockdown of unc79 ^RNAi^ (1471>MB^Gal80^; unc79 ^RNAi^) significantly rescues total sleep compared to γ knockdown of unc79 (1471>unc79^RNAi^, *P* < 0.0432); while, total sleep for γ knockdown of unc79 (1471>unc79^RNAi^) is high compares to other control groups (MB^Gal80^; luc^RNAi^/+, *P* < 0.0001; unc79^RNAi^/+, *P* < 0.0001; 1471/+, *P* < 0.0001; MB^Gal80^; luc^RNAi^/+, *P* < 0.0001; 1471>MB^Gal80^; luc^RNAi^, *P* < 0.0001; and, MB^Gal80^; unc79^RNAi^/+, *P* < 0.0001). Mushroom body ^GAL80^ rescue γ knockdown of unc79 RNAi (1471>MB^Gal80^; unc79 ^RNAi^) restored total sleep to controls (unc79^RNAi^/+, *P* = 0.3715; MB^Gal80^; unc79^RNAi^/+, *P* = 0.7292; and 1471>MBGal80; lucRNAi, *P* = 0.8177). However, 1471>MB^Gal80^; unc79 ^RNAi^*vs* 1471/+ remained significant (*P* < 0.0001). (D) Starvation resistance of mushroom body GAL80 rescue γ knockdown of unc79 RNAi (1471>MB^Gal80^; unc79 ^RNAi^) significantly lower (Gehan-Breslow-Wilcoxon test: χ^2^ = 11.13, *df* = 1, *P* < 0.0009, *N* > 37) compared to mushroom body γ knockdown of unc79 (1471>unc79 ^RNAi^, maroon). All sleep data are violin plots and SR data are survival curves. *P<0.05; ***P < 0.001; ****P < 0.0001.

To determine whether the phenotypes observed are specific lobes of the mushroom body, we tested the effects of *unc79* knockdown in the α/β lobes (*c739*-GAL4), α′/β′ lobes (*c305a*-GAL4), and the γ lobes (*1471* GAL4 drivers, Krashes *et al.* 2007; Aso *et al.* 2009). Flies with *unc7*9 knockdown in α/β lobes and α′/β′ lobes fail to suppress sleep compared to *luc* control, while knockdown in the γ lobes increased total sleep duration compared to *luc* control and fail to suppress sleep when starved (Figure 4A). Starvation resistance is increased in *unc79* knockdown in each mushroom body subtype compared to their respective controls expressing *luciferase*-RNAi (Figure 4B). Therefore, loss of *unc79* function in each subset of mushroom body neurons impacts sleep and metabolic regulation, while selective loss in the γ lobes largely recapitulates the full extent of ubiquitous knockdown. These findings reveal that *unc79* is required in all lobes of the mushroom body for proper sleep and metabolic regulation.

To verify that the increase in sleep and starvation resistance is specific to the mushroom bodies, we examined whether including of the *MB*-GAL80 transgene reverses the effects of *unc79* knockdown in 1471-GAL4 positive neurons. Expression of GAL80 in the mushroom bodies restores sleep and starvation-induced sleep suppression to control levels to flies with *unc79* knocked down in the γ lobes (Figure 4C). Starvation resistance increased in flies with *unc79* selectively knocked down in the (*1471*-GAL4>*unc79*^RNAi^). Blocking expression of GAL4 within the mushroom body restored starvation resistance, and partially restored sleep duration on food, confirming that loss of *unc79* in the mushroom body leads to dysregulated sleep (Figure 4D). In addition, the expression of *MB*-GAL80 restored normal starvation resistance to *1471*-GAL4>*unc79*^RNAi^ flies. These findings validate that the results obtained with *unc79* knockdown using 1471-GAL4 are specific to loss of *unc79* function within the γ-lobe of mushroom bodies.

**Figure 5:**
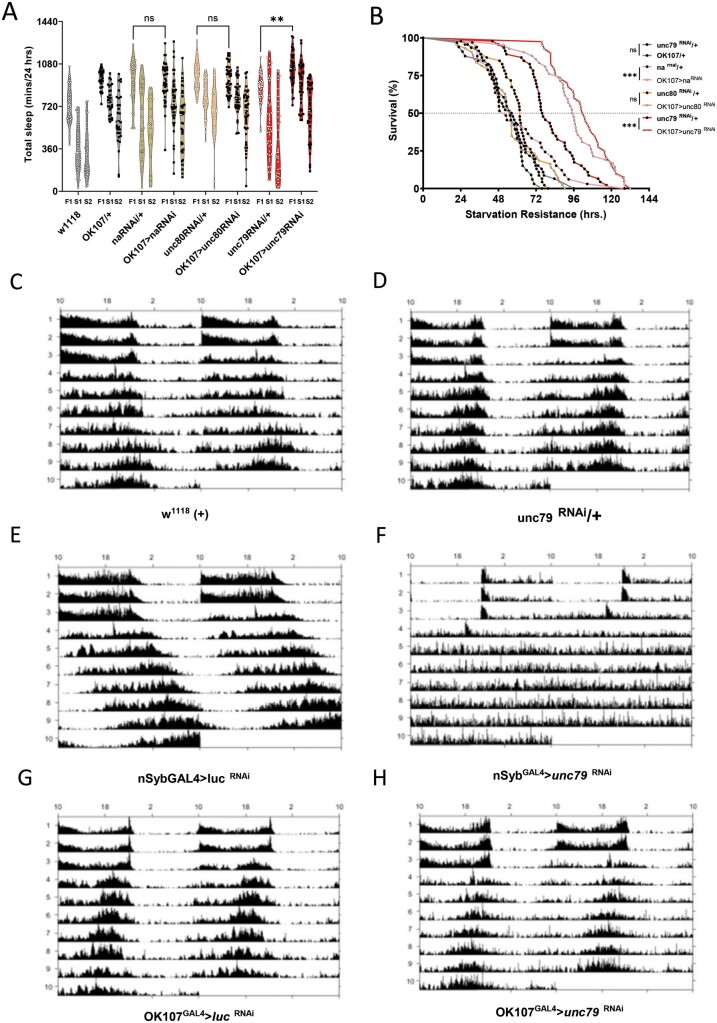
MB knockdown of *unc79* RNAi has normal circadian rhythm. (A) Mushroom body knockdown of *unc79* (OK107>unc79^RNAi^) significantly increased sleep [two-way ANOVA: *F*_(2, 711)_ = 169, *P* < 0.0029, *N* > 40] compared to control (unc79^RNAi^/+). Mushroom body knockdown of narrow abdomen (OK107>na ^RNAi^) did not differ (*P* > 0.9999, *N* > 35) compared to control (*na*^RNAi^/+). Mushroom body knockdown of unc80 (OK107>unc80^RNAi^) did not differ (*P* > 0.9964, *N* > 42) compared to control (unc80 ^RNAi^/+). (B) Starvation resistance mushroom body knockdown of unc79 (OK107>unc79 ^RNAi^) is increased (Gehan-Breslow-Wilcoxon test: χ^2^ = 25.95, *df* = 1, *P* < 0.0001, *N* > 40) compared to control (unc79 ^RNAi^/+). Starvation resistance mushroom body knockdown of narrow abdomen (OK107>na ^RNAi^) is increased (Gehan-Breslow-Wilcoxon test: χ^2^ = 33.54, *df* = 1, *P* < 0.0001, *N* > 42) compared to control (na ^RNAi^/+). Starvation resistance mushroom body knockdown of narrow abdomen (OK107>unc80 ^RNAi^) is no different (Gehan-Breslow-Wilcoxon test: χ^2^ = 2.486, *df* = 1, *P* < 0.1148, *N* > 43) compared to control (unc80 ^RNAi^/+). (C–H) Actogram double plot. Female flies were entrained in light–dark cycle for days 1–3 and dark–dark cycles 4–10 days. Panels (A–C, E, and F) have normal rhythm. Circadian rhythm is disrupted when unc79 is knocked down pan-neuronally, while unc79 knockdown in mushroom body has restored rhythm. All sleep data are violin plots and SR data are survival curves. **P<0.01; ***P < 0.001.

Previous work has revealed that *unc79* functions within the circadian neurons in association with the *unc80* accessory protein and the ion channel *narrow abdomen* (*na*) to maintain locomotor rhythms during constant darkness (Lear *et al.* 2005, 2013; Moose *et al.* 2017). To further investigate whether the sleep and circadian phenotypes are controlled by shared or distinct neural circuits, we knocked down additional components of the *unc79* complex in the mushroom bodies and measured the effects on sleep and starvation resistance. We used RNAi lines from the Vienna *Drosophila* Stock Center, for all lines tested. Knockdown of *unc79* in the mushroom bodies phenocopied the effects on sleep and starvation resistance that were observed with the RNAi line used for screening and genetic mutants, confirming these phenotypes. Knockdown of *na* or *unc80* throughout the mushroom bodies did not increase sleep duration on food or disrupt starvation-induced sleep suppression (Figure 5A). In addition, knockdown of *unc80* and *na* in the mushroom body had little impact on starvation resistance (Figure 5B). These findings raise the possibility that *unc79* functions independently of its canonical complex with *unc80* and *na* to regulate sleep and starvation resistances.

Expression of *unc79* is required within pacemaker neurons to circadian rhythms. Therefore, it is possible that *unc79* functions in distinct populations of neurons to regulate circadian rhythms, from those regulating sleep and metabolic phenotypes. To directly test this possibility, we measured the effects of mushroom body-specific knockdown of *unc79* on free-running activity in entrained animals. As expected, flies with pan-neuronal knockdown are arrhythmic under conditions of constant darkness, while control flies show robust rhythms (Figure 5, C–F). Conversely, knockdown in OK107-expressing cells does not impact circadian activity (Supplementary Figure S4; Figure 5, G and H). Therefore, *unc79* function to regulate circadian rhythms through distinct neural mechanisms that regulate sleep and starvation resistance.

## Discussion

Here, we screened by targeting gene function ubiquitously to identify regulators of sleep and metabolic function. Growing evidence suggests sleep is regulated by complex interactions between the brain and periphery, including the findings that mutants impacting fat storage, and communication from the fat body to the brain significantly impact sleep (Thimgan *et al.* 2010; Slocumb *et al.* 2015; Ertekin *et al.* 2020).

We have identified numerous candidate regulators of sleep, including a novel role for *unc79* in the regulation of sleep and metabolic function. *Unc79* and *unc80* are auxiliary subunits of the sodium leak channel *na*, an ortholog of mammalian *NALCN* family of ion channels (Swayne *et al.* 2009). A number of functions have been identified for this complex including a role in the regulation of circadian rhythms, and anesthesia sensitivity (Lear *et al.* 2005; Humphrey *et al.* 2007). Previous work found that loss of narrow abdomen or *unc79* increased sensitivity to the anesthetics, halothane and isofluorane, and increases sleep (Humphrey *et al.* 2007; Joiner *et al.* 2013) consistent with our findings of increased quiescence in *unc79* mutants. Mutation of *unc79* also facilitates the emergence from anesthesia, raising the possibility that loss of *unc79* promotes state transitions, rather than directly impacting isofluorane sensitivity (Joiner *et al.* 2013). Therefore, suppression of arousal may be involved in anesthesia and sleep (Joiner *et al.* 2013). Mutation of *na* also impacts a number of complex behaviors including social clustering (the distance maintained between individual flies) (Burg *et al.* 2013), and light-mediated locomotor activity (Nash *et al.* 2002). These findings suggest a complex role for *na* and associated *unc79* genes in regulating brain function.

Multiple lines of evidence suggest the role of *unc79* in the regulation of sleep, metabolic regulation of sleep, and starvation resistance is separate from its essential role in regulating circadian rhythms. First, we localize function to the mushroom body, a region that is critical for regulation of sleep and modulation of behavior in accordance with feeding state (Joiner *et al.* 2006; Pitman *et al.* 2006; Sitaraman *et al.* 2015a; Tsao *et al.* 2018). Selective knockdown of *unc79* in the gamma-lobes alone phenocopies *unc79* mutants. While there is little evidence to date for direct involvement of gamma-lobe neurons in sleep regulation, mushroom body output neurons from the gamma-lobes have been found to regulate sleep (Aso *et al.* 2014; Sitaraman *et al.* 2015b). We previously reported that the mushroom bodies are dispensable for starvation-induced sleep suppression, however the manipulations that led to this conclusion involved pharmacological ablation or acute genetic silencing of the mushroom bodies (Keene *et al.* 2010). Therefore, it is possible that loss of *unc79* function impacts sleep circuitry through a mechanism that would not be detected in flies with the previously applied genetic manipulations. Second, *na* and *unc80*, two components of a complex that interacts with *unc79* to regulate circadian rhythms, are dispensable for regulation of sleep and starvation resistance in the mushroom bodies. These findings raise the possibility that *unc79* may function independently of its canonical complex with *unc80* and *na.* However, it is important to note that we are unable to validate the efficacy of the RNAi lines tested, and therefore cannot rule out a possible role for *unc79* functioning through *na.* Further work is needed to address how *unc79* functions may modulate mushroom body physiology and sleep circuitry. Studies examining the role of *unc79* in circadian function and anesthesia sensitivity suggests it functions by regulating *na* activity to modulate neural activity (Moose *et al.* 2017), and it is possible that *unc79* modulates the function of a different ion channel within the mushroom bodies. The structure and function of *na/NALCN* appear to be highly conserved with the channel enhancing neural excitability (Chua *et al.* 2020). Therefore, imaging activity of neurons within the mushroom bodies of flies lacking *unc79* may be informative.

We identify three independent phenotypes to the mushroom bodies. First, we find knockdown of *unc79* in the mushroom bodies promotes sleep suggesting a wake-promoting role for mushroom bodies. The mushroom bodies contain both wake and sleep-promoting neurons, and genetic ablation or silencing of the mushroom body increases wakefulness (Joiner *et al.* 2006; Pitman *et al.* 2006; Sitaraman *et al.* 2015a). It is possible that loss of unc79 is functioning in either sleep promoting or wake-promoting neurons to elicit this phenotype. We also identify two independent metabolic phenotypes to the mushroom bodies. In *Drosophila*, the mushroom circuits have been well-defined including the identification of modulatory neurons, and output neurons that modulate sleep (Aso *et al.* 2014; Haynes *et al.* 2015; Sitaraman *et al.* 2015b). Therefore, the identification of *unc79* as a regulator of sleep provides the opportunity to examine how gamma lobe output neurons are regulated by input neurons and impact the physiology of output neurons.

In addition to the sleep phenotypes, we find *unc79* mutants are resistant to starvation. This finding is particularly interesting because the list of genes chosen for the screen derived from those identified in a Genome Wide Analysis Study for factors associated with starvation resistance (Harbison *et al.* 2004; Hardy *et al.* 2018). Many different factors contribute to starvation resistance including energy stores, basal metabolic rate, and changes in metabolic rate upon starvation. Animals selected for starvation resistance have elevated sleep and do not suppress sleep when starved (Masek *et al.* 2014). Therefore, future work studying starvation selected lines, or other populations of outbred fly lines have potential to identify whether variable expression of *unc79* is associated with naturally occurring differences in sleep and metabolic regulation.

We find that *unc79* most potently impacts sleep and starvation resistance in the gamma lobes, suggesting this population is critical for both sleep metabolic regulation. Output neurons from the gamma lobes have been directly implicated in feeding and fat storage supporting the notion that this region is critical for metabolic regulation (Al-Anzi and Zinn 2018). Future work examining the effects of *unc79* deficiency on the physiology and function of mushroom body output neurons may help identify the role of *unc79* in regulating mushroom body circuits that ultimately regulate behavior and metabolic function. Taken together, these findings add to growing evidence that sleep and metabolic function are integrated. The identification of additional genetic factors that regulate the relationship between sleep and nutritional state through behavioral studies will improve our understanding of the strong associations between sleep loss and metabolism-related diseases. The ubiquitous screen has identified numerous candidate genes that impact sleep, starvation-induced sleep suppression, and starvation resistance, providing candidates that function within and outside of the nervous system. Future study of these genes, such as *unc79*, has potential to advance our understanding of sleep-metabolism interactions and brain-periphery communication.
